# Comparison of Infrared Thermography and Other Traditional Techniques to Assess Moisture Content of Wall Specimens

**DOI:** 10.3390/s22093182

**Published:** 2022-04-21

**Authors:** Letícia C. M. Dafico, Eva Barreira, Ricardo M. S. F. Almeida, Helena Carasek

**Affiliations:** 1 Department of Civil Engineering, Faculty of Engineering (FEUP), University of Porto, 4200-465 Porto, Portugal; up201911110@edu.fe.up.pt; 2 CONSTRUCT-LFC, Department of Civil Engineering, Faculty of Engineering (FEUP), University of Porto, 4200-465 Porto, Portugal; ralmeida@estv.ipv.pt; 3 Department of Civil Engineering, Polytechnic Institute of Viseu, 3504-510 Viseu, Portugal; 4 School of Civil and Environmental Engineering (EECA), Federal University of Goias (UFG), Goiânia 74605-220, Goiás, Brazil; hcarasek@ufg.br

**Keywords:** infrared thermography, surface moisture meter, gravimetric method, moisture, diagnosis

## Abstract

High moisture content is a recurrent problem in masonry and can jeopardize durability. Therefore, precise and easy-to-use techniques are welcome both to evaluate the state of conservation and to help in the diagnosis of moisture-related problems. In this research, the humidification and drying process of two wall specimens were assessed by infrared thermography and the results were compared with two traditional techniques: surface moisture meter and the gravimetric method. Two climatic chambers were used to impose different ambience conditions to each specimen, to evaluate the impact of air temperature and relative humidity in the results. The qualitative analysis of the thermal images allowed the identification of the phenomena. The quantitative analysis showed that the order of magnitude of the temperature gradient that translates high humidity levels is substantially different in the two chambers, pointing to the influence of the surrounding environment. The presented analysis contributes to identifying the criteria indicative of moisture-related problems in two different scenarios and discusses the correlation between the non-destructive techniques and the moisture content in the masonry walls. The limitations and future research gaps regarding the use of IRT to assess moisture are also highlighted.

## 1. Introduction

Moisture is a common pathology in buildings and is complex to diagnose and repair. It leads to problems such as reduction of the building’s durability, reduction of the comfort of the users, decrease of the quality of the indoor environment, and eventual structural damage [1,2,3,4]. Moisture in buildings usually occurs due to the lack of compliance with technical standards and good construction practices, either through the use of low-quality material, lack of details and specifications in the project, inadequate execution or use, and lack of maintenance, or even through a combination of all these factors.

Among the causes of moisture damage, one of the most common is capillary rising damp [5], which results from the prolonged and continuous action of the water existing in the ground, which is absorbed by foundations and pavements and ascends through a permeable structure in a vertical flow [6].

An appropriate diagnosis of the moisture-related pathologies is of major importance not only for repairing the problem, but also for identifying the cause of the flaw, which is important information for preventing its occurrence in the future. In addition to this, the important role that moisture plays on the durability of external walls highlights the need for precise and easy-to-use techniques for monitoring moisture in construction materials, making it possible to characterize the current state of the building and/or the extension of the damage, as well as the rate of deterioration, and, if necessary, to support the selection of the most adequate preventive measures [4,7].

To measure the moisture content, traditional techniques can be used, such as the Gravimetric Method (GM), which consists of collecting samples and weighing them throughout the drying process, and modern techniques, such as Nuclear Magnetic Resonance (NMR), microwave reflection, Infrared Thermography (IRT), or techniques that are based on the electric properties of materials [1], such as the contact moisture detection technique, using a Surface Moisture Meter (SMM).

IRT is a non-destructive test that does not require contact with the surface and allows the assessment of the thermal behavior of existing building materials, enabling the identification of potential problems [2], since defects typically cause a disturbance in the heat flow, which generates temperature differences on the surface of the elements [8].

Based on the above, there is a need to carry out studies to assess the feasibility of using non-destructive tests for diagnosing rising damp in masonry systems. IRT and SMM are very promising techniques, but still do not have their application consolidated and fully clarified, pointing to the need for criteria from non-destructive techniques that are indicative of moisture-related anomalies in masonry. This paper aims to combine the use of IRT and SMM with GM to identify the correlation between these techniques and the feasibility of using the non-destructive tests for diagnosing rising damp in masonry walls. The experimental setup that was prepared also allowed to evaluate the influence of the variation of environmental conditions on the results. Both humidification and drying process were assessed, since, in practice, the water supply and the environmental conditions may vary, causing the wall to experience several humidification/drying cycles throughout its life cycle.

## 2. Use of Non-Destructive Techniques to Diagnose Pathologies in Buildings

The phenomenon of capillarity can be verified when a thin tube is immersed in a container with water, as the water rises inside the tube. In this phenomenon, which is a result of surface tension, molecules close to the surface of the liquid are attracted by the molecules of the glass tube, and there is a curvature of the water along the walls of the tube [9]. The height of capillary rise in a tube can be found using Jurin’s Law, according to Equation (1).

(1) $$h=\frac{2\gamma \cos\theta }{\rho \mathrm{gr}},$$

where h is the capillary rise height (in m), γ is the surface tension (in N/m), r is the capillary radius (in m), θ is the contact angle of the liquid with the glass of the tube (in degrees), ρ is the density of the liquid (in kg/m^3^) and g is the acceleration of gravity (in m/s^2^).

However, in practice, Jurin’s Law cannot be accurately applied to estimate the capillary rise in porous materials, since there are other factors that influence the process [1], such as surface evaporation conditions and the amount of water available at the base of the materials [9,10]. In fact, the rise of water in a wall will progress to the level where the amount of water evaporated is equal to the water absorbed from the soil by capillary forces [11]. The height of the wet front will thus be determined by the interaction between these two variables: if the evaporation rate increases, the wet front lowers, and if the evaporation rate decreases, the wet front rises [12].

To diagnose moisture-related pathologies, techniques such as IRT can be used. In civil engineering, image processing techniques are often used to detect building defects [13] and several pieces of research have been developed to evaluate the applicability of IRT for analysis of cracks in coatings [14,15,16], detection of detachments and voids in concrete structures [17,18,19,20], detection of air infiltration [21], identification of structural elements [22], detection of detachments in coatings [16,23,24,25], and also for detection of moisture [2,3,7,26,27,28,29,30,31,32,33,34].

In theory, IRT can be used to map the moisture distribution and identify areas with atypical moisture content, since changes in moisture content are related to changes in surface temperature [35], according to three physical phenomena: evaporative cooling, reduced thermal resistance, and high heat storage capacity [2].

The phenomenon of evaporative cooling occurs because surface evaporation is an endothermic reaction, which induces a decrease on surface temperature. If a material has a moisture content higher than the humidity of the surroundings, the moisture inside the material will start to evaporate, and this will lead to a decrease in the surface temperature of the material; therefore, regions with high moisture content subject to evaporation will appear on thermal images with a lower temperature than regions without defects [33]. The factors that most influence surface evaporation are: the relative humidity of the air close to the surface, the air temperature, the water content of the material, the physicochemical characteristics of the material, and the content of soluble salts [35,36].

Regarding the phenomenon of reduced thermal resistance, the specific heat and thermal conductivity of water are higher than that of dry building materials. Therefore, when there is water in the pores of these materials, their density, specific heat, and thermal conductivity will increase [36], and consequently, the thermal resistance will be lower. Then, as the heat flux through these wet materials is greater, a heterogeneous thermal pattern will be created, with different surface temperatures between the dry and wet regions [2].

The principle of high heat storage capacity is related to the fact that a wet material will respond more slowly to a change in air temperature than a dry material. Based on this principle, during surface heating, dry areas will heat up faster than wet areas and, with surface cooling, dry areas will also cool faster [2].

Although it is known that changes in moisture content are related to changes in the surface temperature of materials, the use of IRT to assess it is still under development, since it is not really clear whether it is possible to detect moisture before any visible marks occur [2]. Additionally, there are no technical standards for the use of IRT in moisture detection [3]. Among the difficulties of using IRT is the quantitative analysis, aiming to correlate the differences in the surface temperature with the actual moisture content of the wall, due to the complex and variable relationship between evaporative flow and surface temperature [35] and the high number of variables intervening in the phenomenon, such as wind velocity and direction, dirt at the surface, solar radiation, air temperature, relative humidity and different thermal conductivity of inner layers [27].

Some studies have also shown that IRT, if used in combination with other non-destructive techniques, such as the electrical resistance measurement technique or the ground penetration radar technique, can provide good information for a correct and accurate diagnosis of moisture-related problems in buildings [26,37].

The electrical resistance measurement technique allows the detection of moisture content indirectly by measuring the resistance to the passage of an electrical current between two electrodes in contact with the material surface [38]. The value displayed on the moisture meter screen is, however, on a relative scale, indicating the size of the signal, meaning that higher values represent a higher moisture content [27]. Special attention should be paid to the results obtained with moisture meters since soluble salts can affect the readings [39]. Additionally, sometimes the moisture content readings provided by different equipment under the same circumstances can differ significantly [26]. For these reasons, it is necessary to be careful when measuring using this technique, by being aware of the conditions of the inspected substrate and the correct interpretation of the results.

## 3. Materials and Methods

### 3.1. Framework

**Figure 1:**
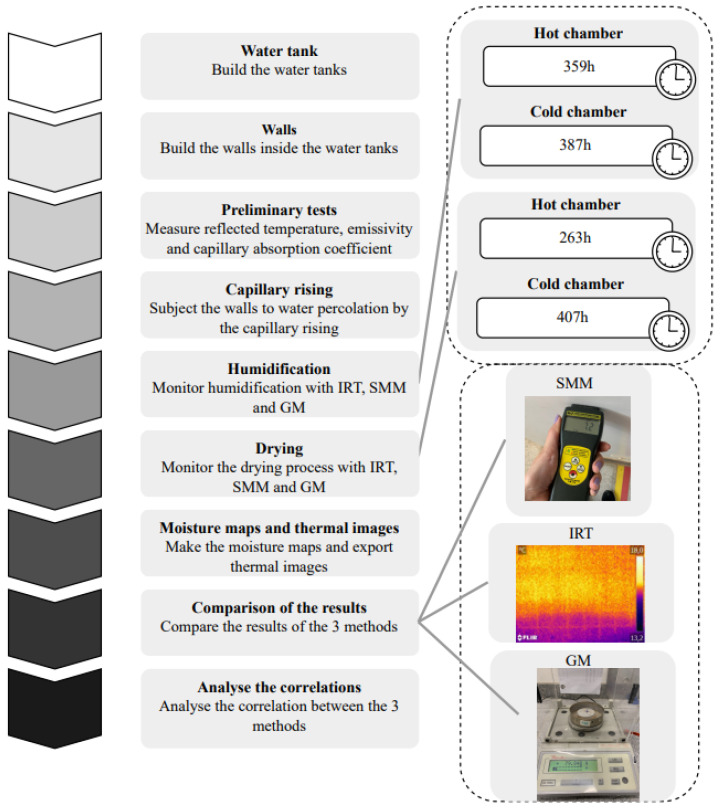
Framework of the laboratory campaign (test procedures and data analysis).

Figure 1 shows the framework of the laboratory campaign carried out for monitoring moisture by capillary rise with Infrared Thermography (IRT), Surface Moisture Meter (SMM), and the Gravimetric Method (GM). It includes not only the experimental procedures, but also the quantitative analysis to test the correlation between these techniques.

**Figure 2:**
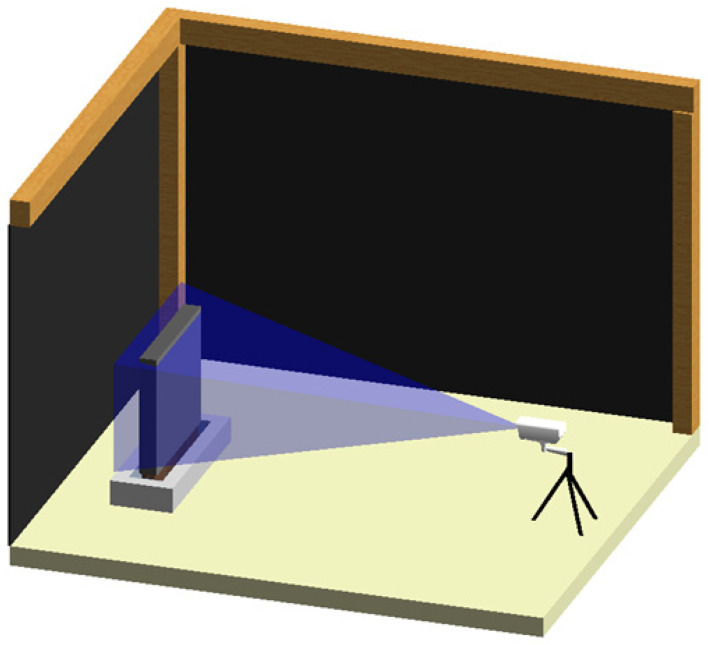
Schematic representation of the test layout.

Firstly, two water tanks were built, each one in a different climatic chamber. After building and waterproofing the tanks, one wall specimen was built inside each tank. Figure 2 shows a schematic representation of the test layout inside the climatic chambers. In each chamber, the humidification of the wall was first assessed on one of the largest sides of the wall and then the wall was rotated 180 degrees and the drying process was assessed on the other side.

In one of the chambers, named in this work the “hot chamber”, the temperature (T) and relative humidity of the air (RH) were set as 30 °C and 60%, respectively. In the other one, named the “cold chamber”, the T and RH were set as 15 °C and 90%, respectively. Due to the size of the climatic chamber, the ambient conditions were not entirely homogeneous. Table 1 contains the minimum, the maximum, and the average values of T and RH obtained in the hot and cold chambers, separately, for the humidification and drying. Within each phase, the ambient conditions present some variability, but the average values are identical in the two phases.

### 3.2. Materials Characterization

The walls used in the experimental campaign were built using hollow ceramic brick, with 9 × 19 × 29 cm^3^, and an industrialized mortar was used both as bricklaying and as rendering. The rendering was applied on the four vertical faces of the walls, with a thickness of 0.02 m. The lower 0.13 m of the walls was not rendered to accelerate the water percolation. The properties of the industrialized mortar are shown in Table 2. The water absorption coefficient of the mortar was measured following the specifications of the standard EN 1015-18 [40], and the remaining properties were collected from the manufacturer’s data sheet. No properties of the brick are presented, since only the mortar was within the scope of this work.

### 3.3. Equipment Characterization

The specifications of the thermographic camera and the SMM used to carry out the tests are shown in Table 3 and Table 4, respectively.

Before carrying out the tests, the thermographic camera was calibrated by the manufacturer. Then, tests to determine the reflected temperature and the emissivity were carried out according to ASTM E1862-97 [43] and ASTM E1933-99a [44], respectively.

Before starting the measurements, it was necessary to select in the SMM the code of the material being analyzed, according to the density of that material, as stated in the manual. The selected density was 1900 to 2000 kg/m^3^. Additionally, according to the SMM manual, there is a coded tricolor LED indicating the moisture status, controlled by 2 limits: 13 and 18. If the measurement is less than 13, the light turns green, if it is greater than 18, the light becomes red. Between the two, it turns yellow. The values given by the equipment are relative; therefore, they do not indicate the actual moisture content of the material: the green light indicates that the moisture on the surface is “impossible”, if it is yellow, it is “possible” and, if it is red, it is “inevitable”. Therefore, these criteria were used for the analyses.

The thermo-hygrometer used to monitor the variation of temperature and relative humidity inside the climatic chamber has a temperature range from −40 °C to 80 °C, with a resolution of 0.1 °C, and a relative humidity range from 0 to 100%, with a resolution of 0.1% [45].

### 3.4. Measurement Procedures

**Figure 3:**
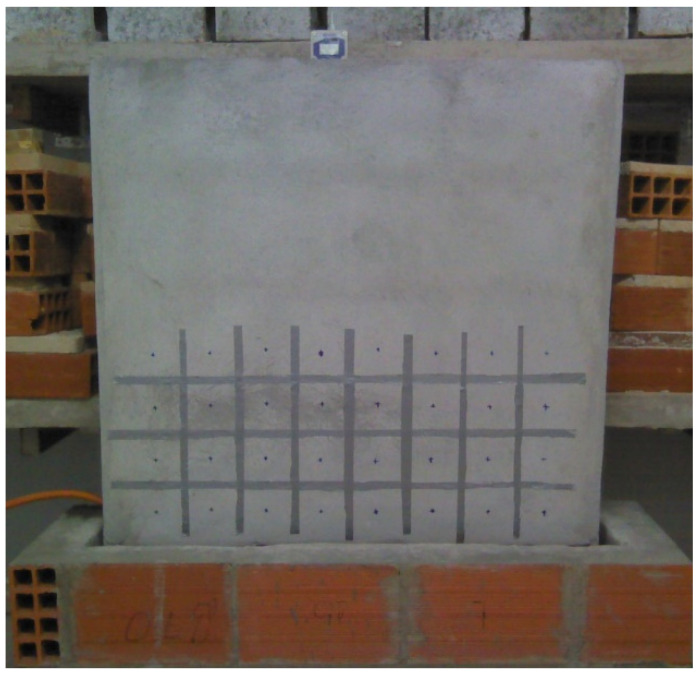
Areas limited on the surface of the wall to assess moisture during the humidification and the drying phases.

Table 5 depicts the timeline for the measurements for each one of the four tests performed. To monitor the humidification and the drying phases of the wall, several areas were marked on its surface, limited by aluminum tape to be detected by the IR camera, as shown in Figure 3. This procedure was necessary, because GM is a destructive method, requiring a clear definition of the location where the sample was collected in each time step. In each test, the first measurement (IRT, SMM and GM) was performed on the left side of the wall, while subsequent measurements were performed on the following intact columns.

**Figure 4:**
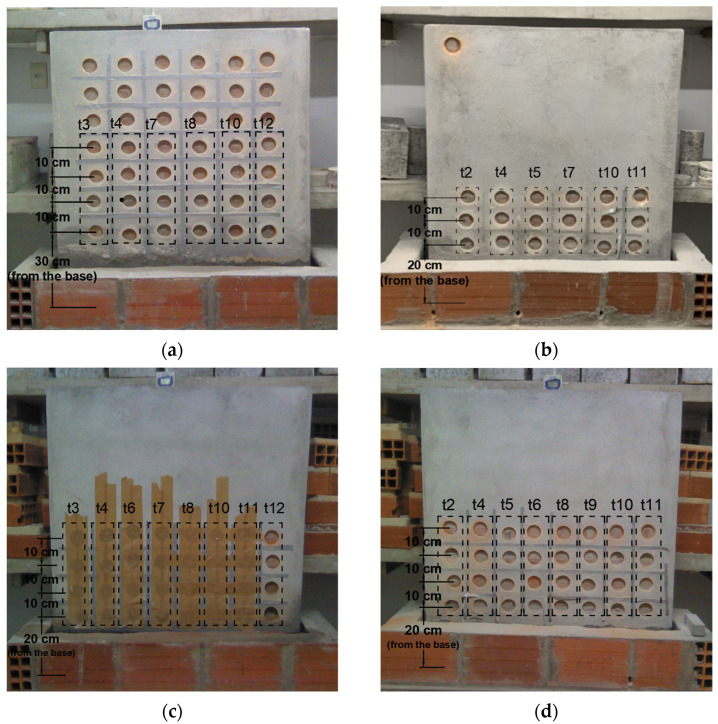
Measurement areas in the humidification and drying of the walls: (**a**) humidification—hot chamber; (**b**) drying—hot chamber; (**c**) humidification—cold chamber; (**d**) drying—cold chamber.

In the humidification process of the wall placed in the hot chamber, 24 areas were selected. For the drying phase, only 18 areas were considered, since the humidification process showed that the water did not reach the fourth row. In the cold chamber, 32 areas were selected, in both the humidification and drying phases. It is noteworthy that in the humidification test of the wall located in the hot chamber, the first row was located 10 cm above the first rows of the other three tests. Additionally, in the tests carried out in the cold chamber, samples from 8 columns were extracted, while in the hot chamber, samples were collected only from 6 columns. These adjustments were necessary, because in the first test that was carried out (humidification of the wall in the hot chamber), the water level did not reach the expected maximum level. Figure 4 shows the measurement areas in the humidification and drying of the walls, and the measurement times in which the samples were extracted with the GM.

The procedure for performing the measurements was as follows:

1. Thermal image of the surface was captured. Only the intact columns were considered in the data analysis of the thermal images.
2. In each measurement time, three measurements were carried out with the SMM in all the remaining undisturbed columns; three measurements were carried out with the SMM to obtain the average of the moisture measurement provided by the equipment. Afterwards, measurements with the SMM were launched in software to generate moisture maps.
3. The samples for the GM were collected from all the areas included in the column of the corresponding time step, using a hole saw 53 mm in diameter. After removal, the samples were placed in metallic bowls, properly identified, and previously weighed. The samples only included the mortar of the rendering of the wall.
4. After collecting the sample and placing it in the bowl, it was weighed and then placed in an oven at a temperature of 105 ± 5 °C. The stabilization of the mass was monitored every 24 h until the difference between subsequent weighing was less than 0.1% of the sample mass.

### 3.5. Criteria for Data Analysis

#### 3.5.1. Thermal Images

**Figure 5:**
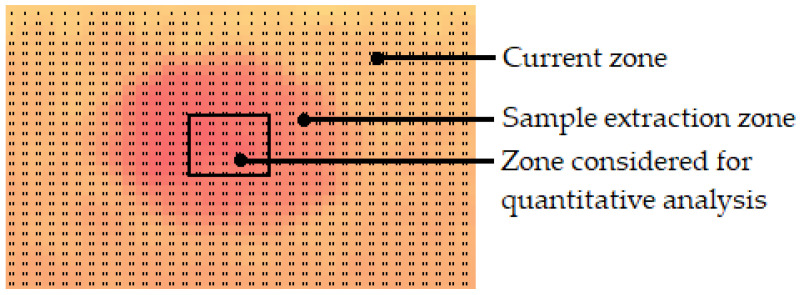
Selected temperatures for the quantitative analysis.

After extracting the matrix of temperatures for each thermal image, the temperatures corresponding to the extraction region for the GM were identified and the 36 central values were selected and averaged for the quantitative analysis (Figure 5). The temperature difference ∆T [°C] was calculated according to Equation (2).

∆T = T_mdry_ − T_txcentral_, (2)

where T_mdry_ [°C] is the average temperature in the “dry region” at the time of measurement (the “dry region” corresponds to the upper half of the whole thermal image of the wall), and T_txcentral_ [°C] is the average temperature of the 36 values at the central positions of the extraction region at the time of the measurement, corresponding to the zone indicated in Figure 5. The ∆T value traduces the magnitude of the temperature difference between the extraction region and the dry region at the time of the measurement.

#### 3.5.2. Moisture Content Using GM

According to the literature, in brick walls with rendering, moisture content above 3% is considered to be high, and above 5% is considered to be damp. In both cases, these limits point to a need to undertake actions to reduce the moisture content in the masonry [46,47]. The reference limits available in the literature were therefore used as the basis to discuss the results obtained by IRT, SMM and GM. In the tests carried out in the hot chamber, values above 3% were considered to be high, while in the cold chamber, this value was adjusted to 4%.

## 4. Results—Qualitative Analysis

**Figure 6:**
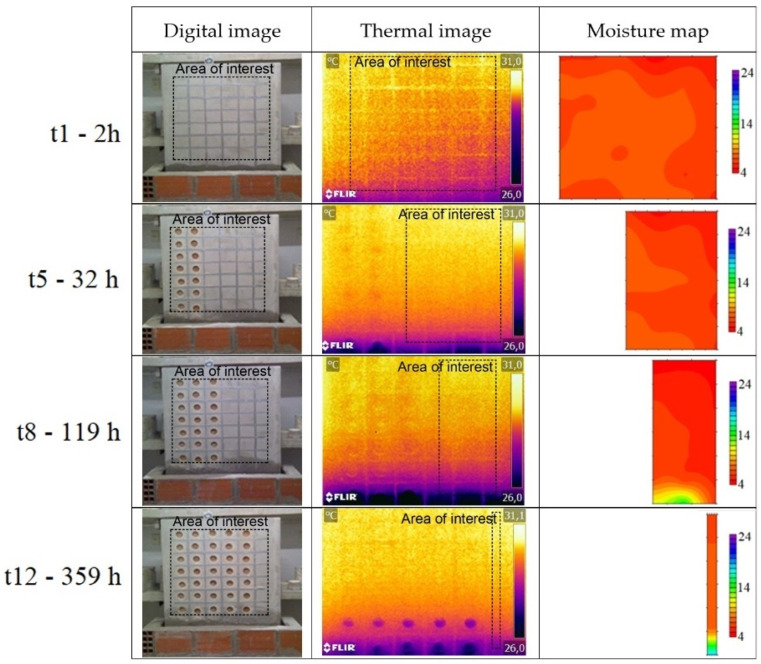
Digital images, thermal images, and moisture maps of the humidification test of the wall in the hot chamber.

**Figure 7:**
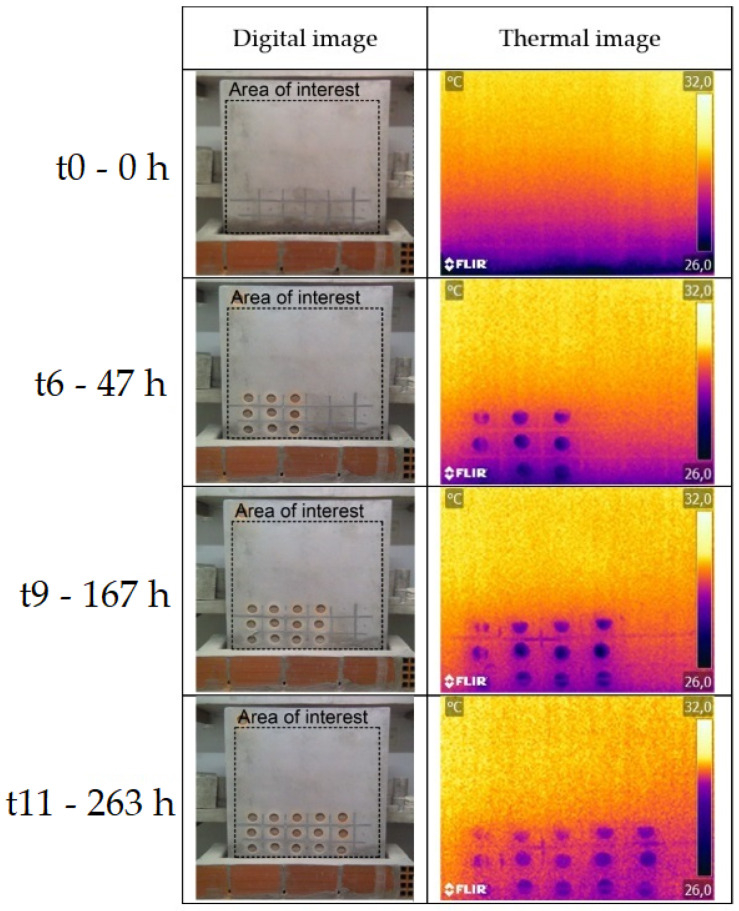
Digital images and thermal images of the drying test of the wall in the hot chamber.

**Figure 8:**
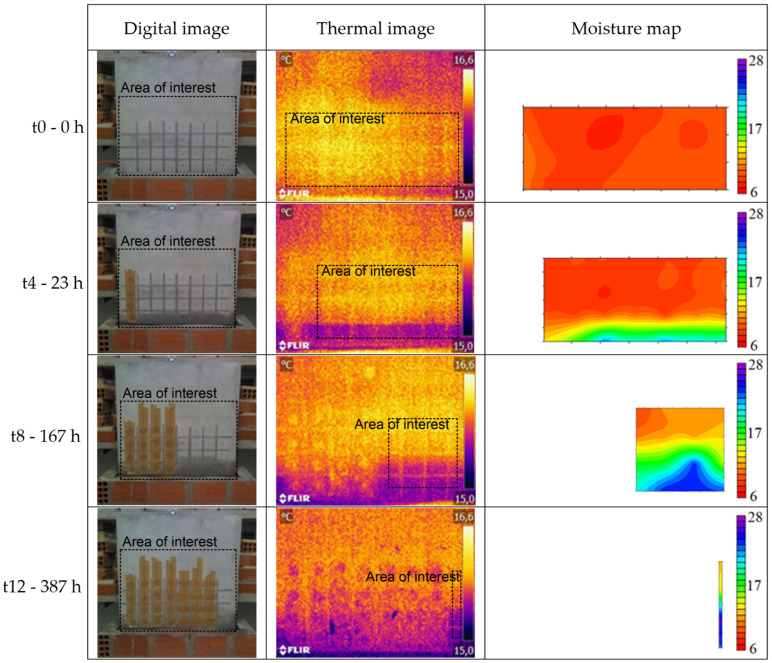
Digital images, thermal images, and moisture maps of the humidification test of the wall in the cold chamber.

**Figure 9:**
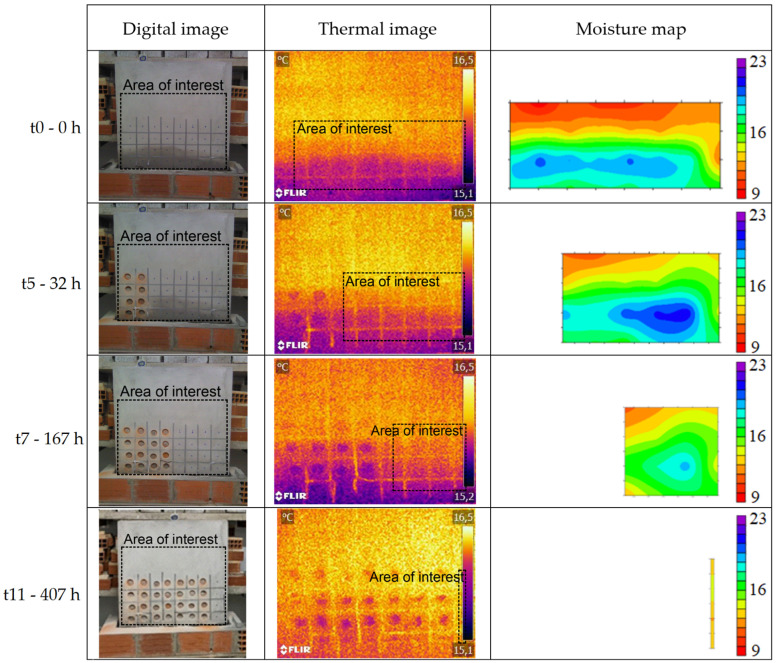
Digital images, thermal images, and moisture maps of the drying test of the wall in the cold chamber.

The qualitative analysis of the results obtained during the humidification and drying tests of the walls, located in the hot and cold chambers, was carried out through the evaluation of the digital images, thermal images, and moisture maps (Figure 6, Figure 7, Figure 8 and Figure 9). The thermal images were taken over the areas demarcated by the rectangles in the digital images. The moisture maps were made considering the area demarcated by the rectangles in the thermal images, which corresponded to the intact columns. Data referring to the SMM in the drying phase of the wall located in the hot chamber and data referring to the first three measurements of the humidification of the wall in the cold chamber had to be discarded due to measurement problems.

The thermal images of the wall in the hot chamber during the humidification phase show that a thermal gradient between the first row and the others can be immediately identified after the beginning of the test, even though the moisture at that location only became visible to the naked eye and on the moisture maps after several days. This phenomenon occurs during all the tests in the hot chamber (humidification and drying), as thermal gradients were identified where moisture was not visible to the naked eye or detected in the moisture maps. Comparing digital images, thermal images, and moisture maps, it is possible to state that in the latter moisture is only detected with some delay.

As for the thermal gradient due to capillary rise, it gets greater with higher temperatures and lower RH. It was also found that this gradient reaches the maximum value after approximately 5 days of humidification. The difference between the thermal gradients (moist area versus dry area) in the two chambers can be explained by the mortar’s drying potential, which is related to the vapor pressure difference between the air and the surface of the specimen. In the hot chamber, this difference is higher, and therefore, there is greater evaporation and, consequently, higher thermal gradients.

## 5. Results—Quantitative Analyses

### 5.1. Thermal Gradient versus Moisture Content

**Figure 10:**
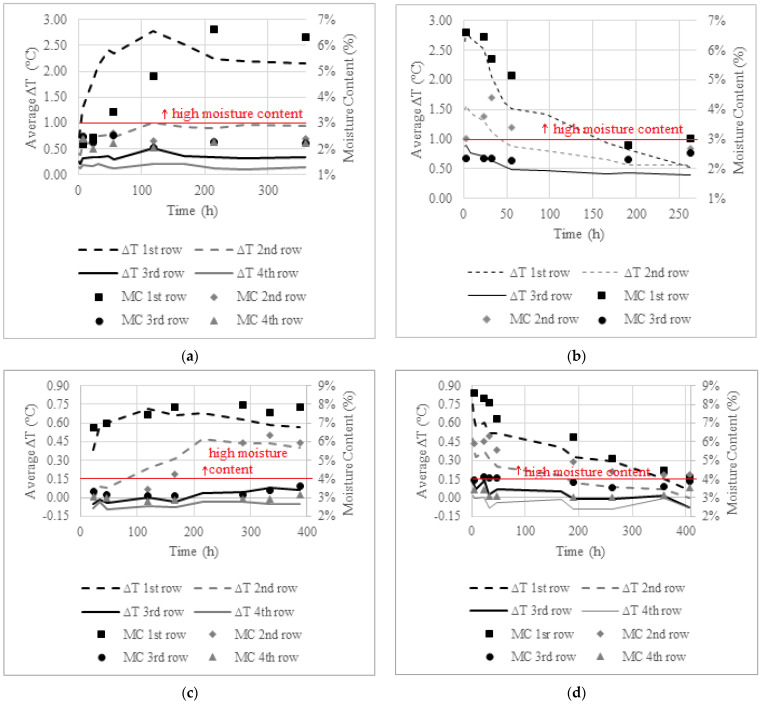
Average ∆T and moisture content (MC): (**a**) humidification—hot chamber; (**b**) drying—hot chamber; (**c**) humidification—cold chamber; (**d**) drying—cold chamber.

The time variation of the thermal gradient (∆T) and the moisture content (MC) by row is shown in Figure 10. The average ∆T was calculated disregarding the areas of the row where the samples were extracted. The value of the MC is the one obtained with the GM in each area, at the measurement time in which the sample was extracted. In Figure 10, the limit for MC in masonry according to the literature is also marked (see Section 3.5.2).

As can be seen in Figure 10a, during the humidification phase in the hot chamber, only a few points with high moisture content were obtained, as the moisture content in rows 2 to 4 was always below 3%. It is also possible to see that the ∆T rises quickly at the beginning of the test, even before the GM indicates high moisture contents. Although IRT identifies the areas with high moisture content, it does not directly quantify how moist they are, since high values of ∆T correspond both to high and low values of moisture content, as can be seen in the first row (t = 56 h and t = 215 h). It is also important to point out that at the end of the test, ∆T in the second row was almost 1.0 °C and the GM did not indicate high moisture content.

Figure 10b, referring to the drying phase in the hot chamber, shows that ∆T decreases more sharply in the first 50 h of drying. In the first row, there is a good agreement between ∆T and the drop of the moisture content measured by the GM. However, in the second row, a slight increase in the moisture content can be seen in the first 32 h, while the ∆T keeps decreasing. In the first 50 h, the points of the third row present ∆T values between 0.5 and 1.0 °C, but the GM does not point to high moisture content in this region. At the end of the test, temperature gradients in the three rows are almost 0.5 °C, and the GM does not indicate high moisture. This situation may be related to the high evaporation capacity of the wall in the hot chamber that promotes evaporation and, consequently, greater thermal gradients even when the values of MC are low. It may also have been influenced by the extraction procedure of the samples in an environment with high temperature and low RH, which may have contributed to decreasing the amount of moisture in the samples. According to Figure 10a,b, in the tests carried out in the hot chamber, the maximum values of ∆T were around 3.0 °C.

Figure 10c refers to the humidification phase in the cold chamber. Negative values can be identified in the third and fourth rows, which may correspond to measurement noise, as those rows had no capillary water at those measurement times. The low ∆T values obtained in this test, of at most 0.70 °C, can be explained by the low drying capacity, as previously explained in the qualitative analysis (see Section 4). The ∆T in the first row stabilized after approximately 150 h of wetting, while in the second row, stabilization occurred after approximately 200 h. Although the values of ∆T obtained in this test are low, they indicate high moisture content, as also shown in Figure 10c.

By analyzing Figure 10d, it is possible to see that ∆T follows the same tendency of the moisture content in all rows, during the entire drying phase. As in the humidification phase, negative values of ∆T, close to 0, were identified, which can also correspond to measurement noise. In this test, even very low ∆T, below 0.20 °C, corresponded to high moisture content, above 4%.

### 5.2. Average SMM Results versus Moisture Content

**Figure 11:**
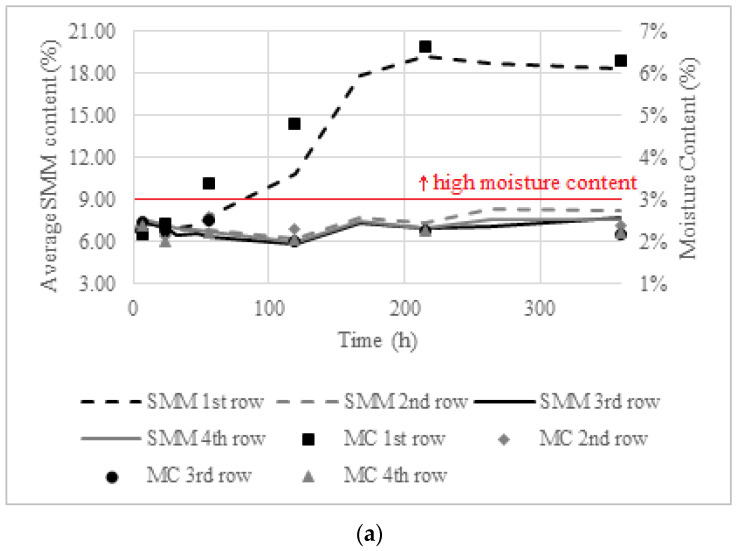

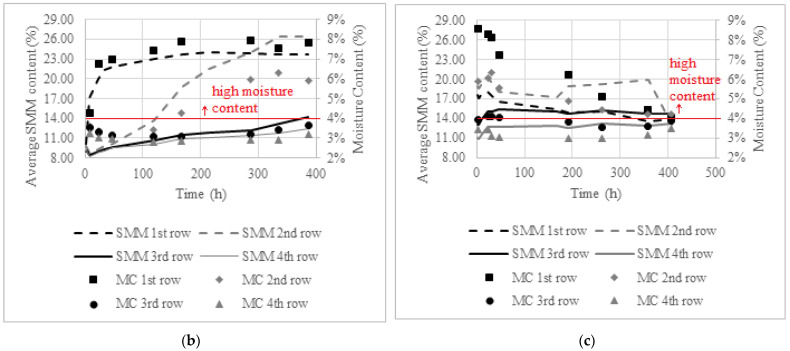
Average SMM results and moisture content (MC): (**a**) humidification—hot chamber; (**b**) humidification—cold chamber; (**c**) drying—cold chamber.

The variation of the SMM results and the MC at each row at each measurement time are shown in Figure 11, which was created following the same methodology used in Figure 10. Figure 11a makes it possible to point out that, in the humidification phase in the hot chamber, both the GM and the SMM indicated moisture only in the first row, although a good agreement was found between the SMM results and the MC.

During the humidification phase of the wall in the cold chamber (Figure 11b), the agreement between the values measured by the SMM and the MC provided by the GM was generally very good in all rows. During the drying phase (Figure 11c), the SMM and the GM presented different results, since throughout the test, the SMM showed higher moisture in the second row, while the GM indicated higher moisture in the first one. There is no reasonable cause for the increase in the SMM values in the first row between 167 to 263 h, which may indicate that the SMM does not have good precision, as it can be influenced by the adjustment of the equipment to the surface and by the pressure in which the measurements are made.

### 5.3. Discussion of the Quantitative Results

**Figure 12:**
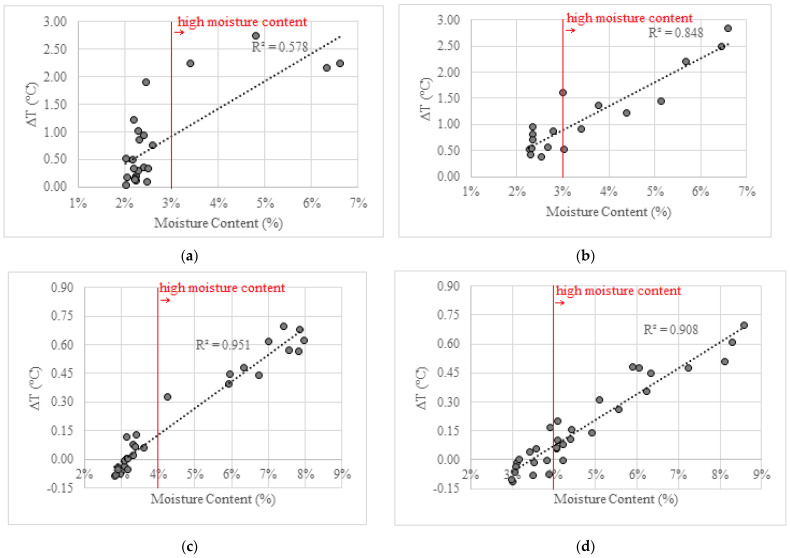
Scatter plot of the ∆T and the moisture content: (**a**) humidification—hot chamber; (**b**) drying—hot chamber; (**c**) humidification—cold chamber; (**d**) drying—cold chamber.

Figure 12 depicts the linear correlation between ∆T and moisture content, obtained by the GM, for all the tests performed. As shown in Figure 12a, during the humidification inside the hot chamber, high moisture content corresponds to ∆T higher than 2.0 °C. In this test, several measurements occurred in which a ∆T over 1 °C corresponded to a moisture content below 3%. All the measurements with a ∆T below 1 °C presented low moisture content, between 2.0% and 2.5%. For the drying process (Figure 12b), some differences can be noted, as most of the measurements with ∆T over 1 °C corresponded to high moisture content. Moreover, there is a higher linear correlation between the two parameters, as the determination coefficient is 0.85.

In the tests carried out in the cold chamber (Figure 12c,d), all the measurements with ∆T above 0.30 °C indicated high moisture content, above 4.0%. During the drying process, several measurements with very low ∆T, below 0.20 °C, corresponded to high moisture content, between 4.0% and 5.0%. A good agreement between ∆T and moisture content can be identified, both during the humidification and drying phases.

Overall, the results obtained in the humidification test in the hot chamber (Figure 12a) show a different pattern from the others, where the linearity between the two variables is clearly more visible. This phenomenon is probably due to the fact that the height of the first level of the extraction of the samples changed, being higher in this test. This situation conditioned the results, leading to the loss of intermediate data and thus preventing a detailed characterization of the humidification process.

The results also show that the order of magnitude of the temperature gradient that translates into high humidity levels is substantially different in the two chambers. This aspect is especially relevant, because it means that, although this indicator can be important for characterizing high moisture content in walls, it is still not possible to directly correlate it to the exact moisture content. In this sense, it is suggested that in future works, an indicator is developed that also includes the effect of ambience, allowing the generalization of the technique to identify moisture risk areas in walls.

## 6. Conclusions

Moisture analysis with IRT and GM pointed to a great influence of T and RH conditions on the results. In the humidification phase of the wall inside the hot chamber, thermal gradients above 2.0 °C indicated areas with high moisture content, while in the drying phase, the limit decreased to 1.0 °C. The maximum thermal gradient identified in this chamber was around 3.0 °C. As for the cold chamber, in the humidification phase, thermal gradients from 0.3 °C indicated regions with high moisture content, while in the drying phase, even very small thermal gradients, below 0.2 °C, occurred in regions with moisture content. In this chamber, the maximum thermal gradient identified was 0.7 °C. The difference in the thermal gradient between the hot and the cold chamber is due to the higher evaporation in scenarios with high T and low RH and points to the need to include the effect of ambience, allowing the generalization of the technique.

Regarding the variation in ∆T, GM and SMM content during the tests, the following was observed:

1. The ∆T increases in the first rows even before the GM indicates moisture in these areas. Additionally, throughout the humidification and drying phase in the hot chamber, a thermal gradient was identified in regions in which the GM showed no high moisture content;
2. The SMM takes longer to indicate high moisture levels than IRT. In the humidification of both walls, the SMM started to indicate moisture only when it was already visible to the naked eye, after the GM had indicated high moisture content. However, in the drying phase, the SMM continued to indicate areas as having high moisture content even after no signs of moisture were visible to the naked eye;
3. After approximately 5 days of testing, the ∆T reached maximum values in the first row and remained stable or slightly decreased until the end of the test;
4. The SMM values in humidification increase until approximately 10 days of testing, at which point they stabilize, following the MC, which also stabilizes approximately from the 10th day on.

The order of magnitude of the temperature gradient that translates high humidity levels was substantially different in the two chambers, which means that it is still not possible to directly correlate it to the exact moisture content. In future works, an indicator must be developed to also include the effect of ambience, allowing the generalization of infrared thermography to identify moisture risk areas in walls. Moreover, future research can be carried out to define other metrics and to evaluate the accuracy of the use of IRT and SMM to assess moisture in masonry walls. The definition of the required period for monitoring moisture-related defects with these techniques is also a future challenge.

## Figures and Tables

**Table 1:** Environmental conditions in the hot and cold chambers.

Chamber	Phase	Minimum T (°C)	Maximum T (°C)	Average T (°C)	Minimum RH (%)	Maximum RH (%)	Average RH (%)
Hot	Humidification	28.1	36.0	30.3	40	66	61
	Drying	28.2	35.5	30.0	46	67	61
Cold	Humidification	12.8	15.8	14.9	74	92	88
	Drying	13.4	16.3	15.2	77	92	90

**Table 2:** Properties of the industrialized mortar used in the walls.

Fresh bulk density	1600 to 2000 kg/m^3^
Tensile strength in bending	1.5 to 2.7 MPa
Compressive strength	4.0 to 6.5 MPa
Water retention	80 to 90%
Incorporated air content	15 to 18%
Water absorption coefficient	0.50 kg/(m^2^·min^0.5^)

**Table 3:** Infrared camera specifications [41].

Thermal sensitivity	<0.07 °C to 30 °C (86 °F)/70 mK
Detector type	Focal plane array (FPA), uncooled microbolometer
Spectral range	7.5 a 13 µm
IR Resolution	160 × 120 pixels
Minimum focus distance	0.40 m
Field of view (FOV)	25° × 19°
Spatial resolution (IFOV)	2.72 mrad

**Table 4:** SMM specifications [42].

Measurement range	0–80%
Measurement depth	Search/Density mode—20 mm Pin mode—10 mm
Operating conditions	Temperature: 0–50 °C Humidity: below 90% RH
Alarm limits (AL)	AL < 13%—decay impossible 13% ≤ AL ≤ 18%—decay possible AL > 18%—decay inevitable

**Table 5:** Timeline for the measurements for each test.

	Hot Climate Chamber		Cold Climate Chamber	
Time	Humidification	Drying	Humidification	Drying
t0	0 h	0 h	0 h	0 h
t1	2 h	1.5 h	1 h	2 h
t2	4 h	3 h	3 h	4 h
t3	7 h	8 h	8 h	8 h
t4	23 h	23 h	23 h	23 h
t5	32 h	32 h	33 h	32 h
t6	47 h	47 h	47 h	47 h
t7	56 h	56 h	119 h	167 h
t8	119 h	95 h	167 h	191 h
t9	167 h	167 h	215 h	263 h
t10	215 h	191 h	287 h	359 h
t11	263 h	263 h	335 h	407 h
t12	359 h	-	387 h	-
